# Clinical Cases from Practice

**Published:** 1868-09

**Authors:** N. S. Davis

**Affiliations:** Professor of Practical and Clinical Medicine, in Chicago Medical College


ARTICLE XXXVII.

### CLINICAL CASES FROM PRACTICE.

BY N. S. DAVIS, M.D., Professor of Practical and Clinical Medicine, in Chicago Medical College.

*Vaginismus.*—Mrs. H., with her husband, from a distant part of the State, consulted me on the 17th of March, 1868. She stated that she had been married over three years, and, though enjoying fair general health, and feeling the usual sexual desires, had never been able to allow sexual intercourse with her husband, on account of the *terrible* suffering that every attempt had produced.

On attempting to make a vaginal examination with the index finger, it was found necessary to bring her fully under the influence of chloroform before it could be completed. The sphincter at the entrance of the vagina contracted as closely around the finger as the sphincter ani generally does; and without the chloroform, she shrank with terror as the finger separated the labia. Finding no malformation of the parts beyond the entrance of the vagina, except an apparent shortness of the vagina and smallness of the neck of the uterus, she was advised to undergo an operation, such as has been recommended by Dr. J. Marion Sims. This, she and her husband readily assented to; and, on the following day (March 18th), having had the bowels freely moved, so that the rectum should be empty, I proceeded with the operation, assisted by Dr. S. A. McWilliams and two students. The patient was brought into a state of complete anæsthesia by the cautious use of chloroform, and then placed in the same position on the table as for the performance of lithotomy. The labia were separated by the assistants and a portion of the mucous and sub-mucous tissue, corresponding with the original attachments of the hymen, was dissected away with the scissors and forceps, and two incisions were made in the posterior part of the vagina; each commencing about two inches up, one to the right and the other to the left, making them converge into one on the central line posteriorly, and then extending as one incision outward to the edge of the perineum. The incisions, when completed, resembled in shape the letter Y, and were deep enough to sever the dense, sub-mucous cellular tissue, and the fibres of the sphincter vagina muscle. The operation was completed by placing a soft sponge in the vagina and removing the patient into her bed. The hemorrhage was inconsiderable, and under the influence of a moderate dose of morphine, the patient rested comfortably for several hours. The next morning the sponge was removed, the vagina washed out with water, a small-sized vaginal dilator inserted, and allowed to remain five minutes. From this time, the dilator was inserted every day during the first week, allowing it to remain from fifteen to thirty minutes. During the second week, one of a larger size was used in the same manner. During the third week, the dilator was introduced only every second day, one of medium size entering the vagina with but little pain or resistance.

On the 15th of April, the treatment was discontinued and the patient permitted to return home. The incisions had entirely healed, and a letter, received from her husband soon after, announced that all serious obstruction to sexual intercourse had been removed. The lady was again in the city, a few days since (Aug. 21st), and assured me that the operation had been entirely successful.

*Fissures of the Rectum.*—June 6th, 1868, was called to Mrs. L., residing in this city. She was pale, moderately emaciated, nervous system very excitable, appetite impaired, and bowels inclined to constipation. For more than one year she had suffered some from leucorrhœa, and terribly from acute lancinating pain in the anus every time she had a discharge from the bowels. This pain was so acute and protracted after the discharge, that it often induced slight spasms or muscular twitchings, and rendered the patient incapable of leaving her bed for twelve or eighteen hours after each passage. Indeed, the dread of the suffering had induced her to postpone intestinal discharges for eight or ten days at a time.

She had been told, by a previous medical attendant, that the difficulty was occasioned by a tumor growing in the posterior walls of the uterus or in the recto-vaginal septum. Putting the patient under the influence of chloroform, a careful examination was made, both rectal and vaginal. No tumor was discovered, not even a hemorrhoidal one; but just within the sphincter ani were no less than three well-characterized *fissures*, and a tender, abraded appearance of the mucous membrane over nearly the whole circumference of the anus. An operation for the cure of the fissures was advised and readily assented to.

Accordingly, the following day, the patient was placed on the table, and, after I had put her under the full anæsthetic influence of chloroform, Dr. S. A. McWilliams proceeded to make a free incision through the bottom of each fissure, and, in addition, to stretch, moderately, the fibres of the sphincter ani muscle. The patient was placed in bed, and, as the effects of the anæsthetic passed off, an anodyne was given, sufficient to insure rest for the succeeding few hours. After the first two days the intestinal discharges became regular, and were accompanied by only the slight, smarting pain caused by the incisions. The latter rapidly healed, and there has been no return of the previous suffering. The general health of the patient has also much improved.

The chief object in relating this case, is to illustrate the necessity of making sufficiently careful examinations of patients suffering under chronic maladies, to understand their true nature before the patient suffers months, and sometimes years, from ailments that are easily removed.

